# The cement-bone bond is weaker than cement-cement bond in cement-in-cement revision arthroplasty. A comparative biomechanical study

**DOI:** 10.1371/journal.pone.0246740

**Published:** 2021-02-11

**Authors:** Marcin Ceynowa, Krzysztof Zerdzicki, Pawel Klosowski, Maciej Zrodowski, Rafal Pankowski, Marek Roclawski, Tomasz Mazurek

**Affiliations:** 1 Department of Orthopedic Surgery, Medical University of Gdańsk, Gdańsk, Poland; 2 Faculty of Civil and Environmental Engineering, Gdansk University of Technology, Gdańsk, Poland; University of Zaragoza, SPAIN

## Abstract

This study compares the strength of the native bone-cement bond and the old-new cement bond under cyclic loading, using third generation cementing technique, rasping and contamination of the surface of the old cement with biological tissue. The possible advantages of additional drilling of the cement surface is also taken into account. Femoral heads from 21 patients who underwent a total hip arthroplasty performed for hip arthritis were used to prepare bone-cement samples. The following groups of samples were prepared. A bone—cement sample and a composite sample of a 6 weeks old cement part attached to new cement were tested 24 hours after preparation to avoid bone decay. Additionally, a uniform cement sample was prepared as control (6 weeks polymerization time) and 2 groups of cement-cement samples with and without anchoring drill hole on its surface, where the old cement polymerized for 6 weeks before preparing composite samples and then another 6 weeks after preparation. The uniaxial cyclic tension-compression tests were carried out using the Zwick-Roell Z020 testing machine. The uniform cement sample had the highest ultimate force of all specimens (*n* = 15; *R*_*m*_ = 3149 N). The composite cement sample (*n* = 15; *R*_*m*_ = 902 N) had higher ultimate force as the bone-cement sample (*n* = 31; *R*_*m*_ = 284 N; *p* <0.001). There were no significant differences between composite samples with 24 hours (*n* = 15; *R*_*m*_ = 902 N) and 6 weeks polymerization periods (n = 22; *R*_*m*_ = 890 N; *p* = 0.93). The composite cement samples with drill hole (n = 16; *R*_*m*_ = 607 N) were weaker than those without it (n = 22; *R*_*m*_ = 890 N; p < 0.001). This study shows that the bond between the old and new cement was stronger than the bond between cement and bone. This suggests that it is better to leave the cement that is not loosened from the bone and perform cement in cement revision, than compromising bone stock by removal of the old cement with the resulting weaker cement-bone interface. The results support performing cement-in-cement revision arthroplasty The drill holes in the old cement mantle decrease cement binding strength and are not recommended in this type of surgery.

## Introduction

The cement-in-cement revision hip arthroplasty is a well-established method of revising cemented femoral stems [1–6]. It is generally reserved for patients with aseptic loosening of the femoral stem with an intact and well-fixed cement mantle. It is also used for humeral stem revision in shoulder arthroplasty [7–9]. Less commonly, it is used for acetabular component revision [4], two-stage revisions for infected total hip replacements [10] or type B periprosthetic fractures [11]. In general, in patients operated for aseptic loosening [6, 12–17] or fractures [11] the results are promising, but disappointing for septic loosening [10].

The advantages of cement-in-cement revisions include shorter operative time, smaller blood loss and lower risk of intraoperative femur fractures [3, 15, 18]. The patients have to be carefully selected and operative technique has to be strictly followed [1, 4, 5, 16, 19]. Intraoperatively, the remaining cement mantle is examined and if any loosening or damage is encountered, the old mantle has to be removed and an alternative procedure be performed [1]. If the cement mantle is intact, its inner surface is prepared with a rasp, burr or ultrasound device. It is then meticulously cleaned of blood, bone marrow and debris, since any contamination may compromise the bond [1, 20, 21]. The new cement is then introduced in its liquid state to promote integration with the old cement [3, 5, 21]. The new femoral stem should better be slimmer than the original to provide space for the additional new cement mantle [14, 19, 22].

Several biomechanical studies in the past have been performed to analyze the bond between the old and the new cement [1, 23] in cement-in-cement revisions. Generally the results support this kind of revision, provided some technical aspects are followed: preparation of the old cement surface with a rasp [21, 23], careful cleansing of the contaminating tissues [20, 21] and introduction of cement in its liquid phase [3, 24].

Until now, only one biomechanical study investigates a relative strength of the cement-in-cement and bone-cement interface. It concluded that the bone-cement bond after revision with the old cement removal is weaker than the old-new cement interface, thus favoring cement-in-cement revisions [25]. Another study showed that the bone-cement interface shear strength is significantly reduced when the original cement mantle is removed during revision arthroplasty and new cement is introduced into the femoral medullary cavity [26]. The comparison between primary bone-cement bond and the old-new cement bond has not been investigated before. All previous studies have some technical aspects that may be criticized: the use of first and second generation cementing technique [1, 20, 24, 25], disregarding contamination of the cement samples with blood, marrow and debris [21, 23, 24] as well as too short polymerization period of the old cement [1, 25]. Moreover, all previous studies have been tested under uniformly growing loading, whereas cyclic loading would better resemble the actual in vivo loading [1, 27]. The additional drilling of the old cement mantle that may be used to supplement the bond in acetabular revision has also not been investigated yet [4].

The aim of this study is to compare the strength of the native bone-cement bond and the old-new cement bond under cyclic loading, using third generation cementing technique, rasping of the surface, as well as the contamination of the surface of the old cement with subsequent clearing. The possible advantages of additional drilling of the cement surface was also taken into account.

## Materials and methods

Femoral heads were retrieved from 21 consecutive patients (11 female, 10 male) who underwent a standard total hip arthroplasty performed for hip arthritis between 1. March and 21. December 2018 in the Department of Orthopedic Surgery, Medical University of Gdansk, Poland. Mean age was 68 years old (SD = 9.9, min = 54, max = 87). The femoral head retrieval did not affect decision making in their treatment and did not change the course of the surgical procedure. All patients gave their informed written consent to participate in the study. Patients with suspected metastatic neoplasms, femoral neck fractures, rheumatoid arthritis or any other condition that could affect bone quality were excluded from the study. This study was approved by the Independent Bioethics Committee at the Medical University of Gdansk, Poland (issued 21.05.2018, NKBBN/228/2018).

The Biomet Plus Bone Cement was used (Zimmer Biomet, Warsaw, IN, USA). It was prepared with third generation cementing technique, using the Zimmer Biomet Compact Vacuum Cement Mixing System. For all samples, the cement was attached under pressure and kept pressurized for 10 minutes as the operative technique requires.

### Bone—Cement samples

The femoral heads were cut fresh to form cylinders of 3 cm of side length and 18 mm in diameter. On average, two samples were retrieved from every femoral head. The side that was cut from the subchondral bone from the center of the head was then marked, to make sure that every cement block was attached to the same part of femoral head subchondral bone. The bone samples were then frozen and stored for future examination.

**Fig 1:**
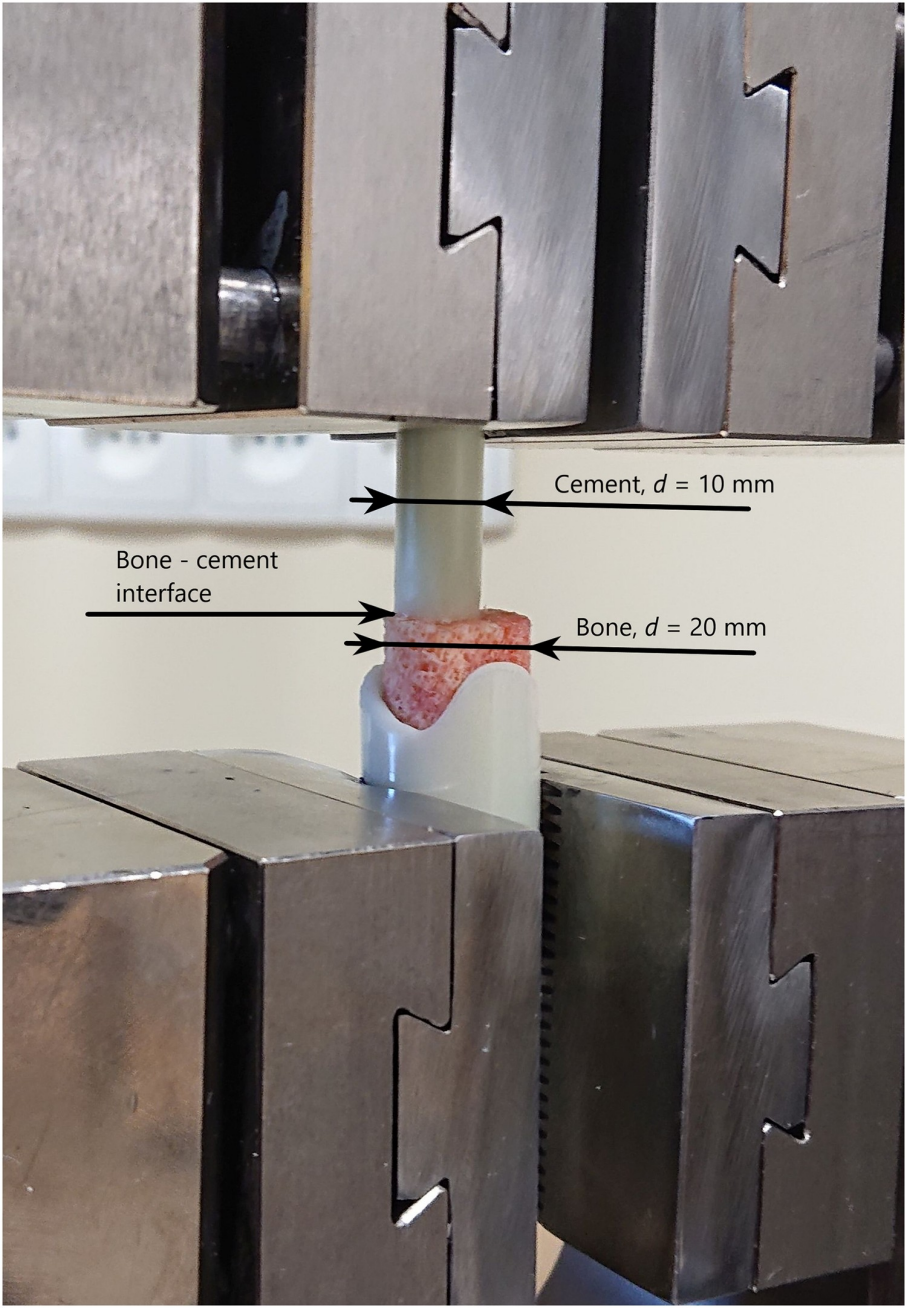
The bone—Cement sample fixed in the testing machine clamps.

The bone samples were then defrosted to room temperature and cleared from marrow with a standard pulsatile jet lavage. Next, the bone samples were embedded in methylmethacrylate to allow proper clamping by grips of the testing machine (Fig 1).

The cement cylinders were then attached under manual pressure to the previously marked bone surface using a custom-made aiming device to ensure straight sample preparation using standard 2 cm^3^ injection syringes as forms for the cement. This preparation was performed by the same investigator (MC) in every case with the attempt to apply similar pressure to every sample. After preparation the samples were stored in cool in a refrigerator (4°C) to avoid bone decay before testing. The samples were tested in the testing machine 24 hours after preparation (Fig 1).

### Uniform cement samples

The uniform cement cylinder was formed using the standard 10 mm diameter, 2 cm^3^ volume injection syringes glued one to another to extend the length of the sample.

### Cement-cement samples

The composite cement cylinder was then prepared. The first part was formed using the standard syringe as a form and then the samples were stored in room conditions for 6 weeks to allow final cement polymerization.

**Fig 2:**
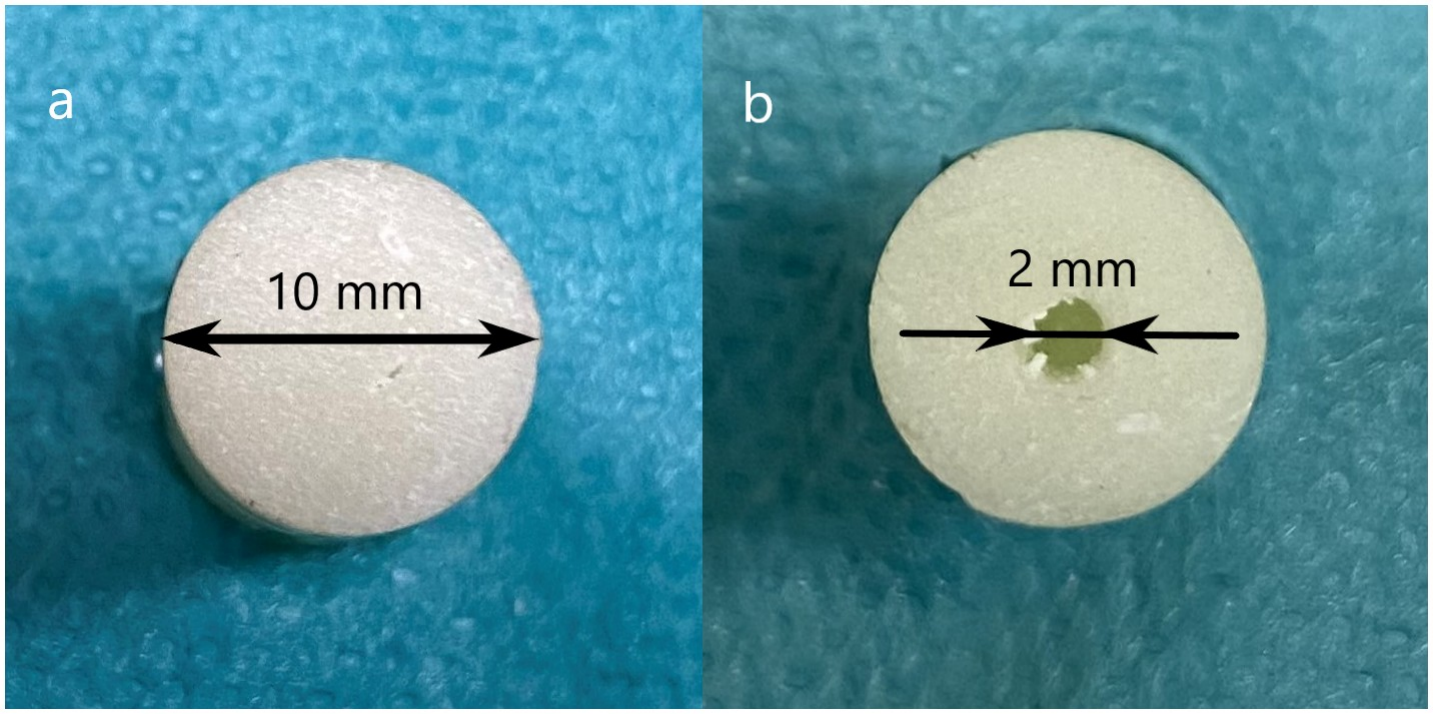
The attachment surface of the old cement after rasping and drilling of the hole. a. The sample surface without drill hole. b. The sample surface with drill hole.

Then, one end of the cylinder was roughened with a rasp. Two groups of cement blocks were prepared from the 6 week old samples: in one group the surface was left intact (*n = 22*, Fig 2 a), in another group (*n = 16*), a hole 2 mm in diameter and 2 mm deep was drilled to allow better cement attachment by increasing of the contact area (Fig 2b).

**Fig 3:**
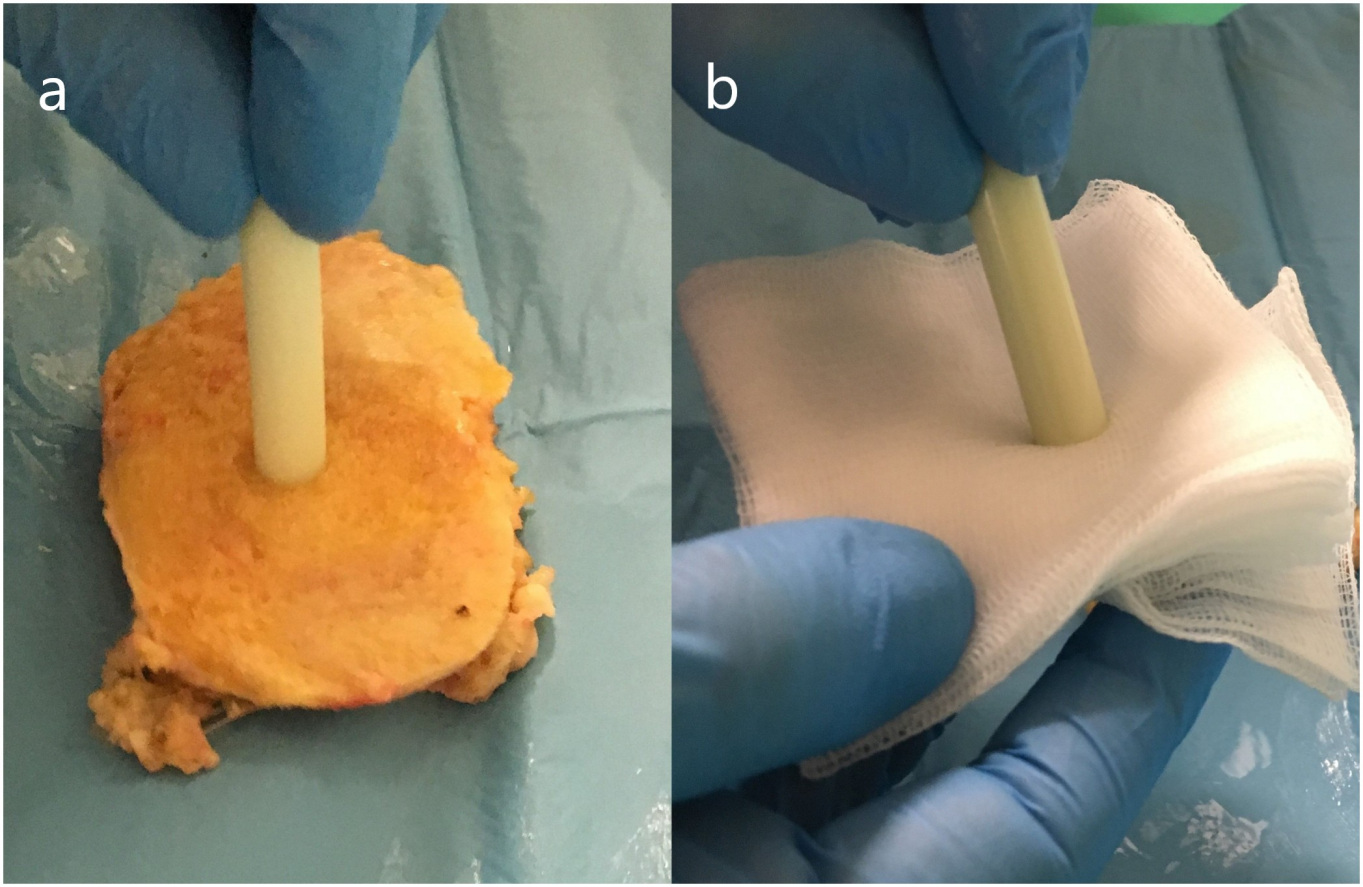
The contamination and clearing of the attachment surface of the old cement sample. a) Contamination with bone marrow. b) Clearing of the contamination with a gauze.

The attachment surface was contaminated with a fresh bone sample that was retrieved during standard arthroplasty to cover the surface with blood, bone marrow and fat, and then wiped with saline soaked gauze, to resemble as closely as possible the intraoperative condition of the old cement mantle (Fig 3a, 3b).

**Fig 4:**
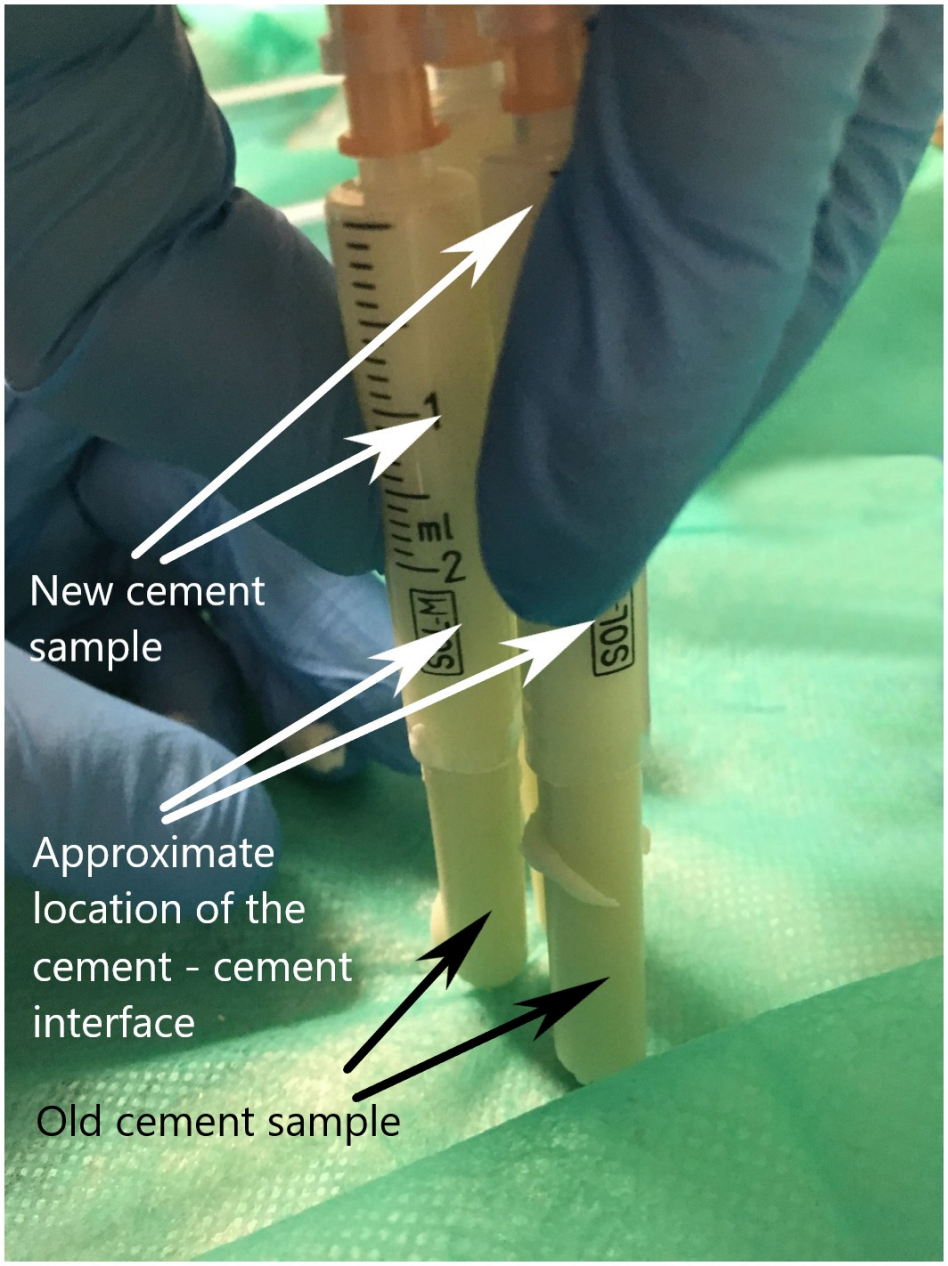
The attachment of the new cement sample.

Then, the new cement cylinder was attached After the syringe was filled with cement the old cement sample was attached under manual pressure to the new cement (Fig 4).

A group of samples without the drill hole (*n = 15*) were tested within 24 hours from preparation to ensure similar polymerization time of the new cement to the bone—cement samples. The remaining composite samples were left for another 6 weeks to allow cement polymerization.

The diameter of the cement samples was *d = 10 mm* (radius *r = 5 mm*), therefore the contact surface *S*_*1*_ in composite samples was *S*_*1*_ = πr^2^ = *3*.*14 x 5*^*2*^ *= 78*.*54 mm*^*2*^.

The drill hole was *d = 2 mm* in diameter (*r = 1 mm*) and 2 mm in depth (*h = 2 mm*). The increased contact surface *S*_*2*_ was the outer surface of a cylinder.

$$S_{2}=\;2\pi rh\;=\;(2\;x\;3.14\;x\;1\;x\;2)\;=\;12.57\;mm^{2}$$

The total contact surface *S*_*3*_ of the drilled samples was *S*_*3*_ *= S*_*1*_ *+ S*_*2*_ *= 78*.*54 mm*^*2*^ *+ 12*.*57 mm*^*2*^ *= 91*.*11mm*^*2*^.

The estimated increase of contact surface for the drilled versus undrilled samples was approximately *x* = 16% (*x* = *S*_2_/*S*_1_·100%). However, the contact surface of this sample became three-dimensional and calculation of the surface just including its increase by the hole may be an oversimplification. The surface of the cancellous bone, as well as the cement surface that is roughened with a rasp, is also three-dimensional, but microscopically. Therefore, the contact surface of the composite sample parts should be considered to be plain, disregarding any surface irregularities in calculations, and the drilling of a hole should be treated as surface preparation similar as roughening of the cement surface with a rasp.

**Fig 5:**
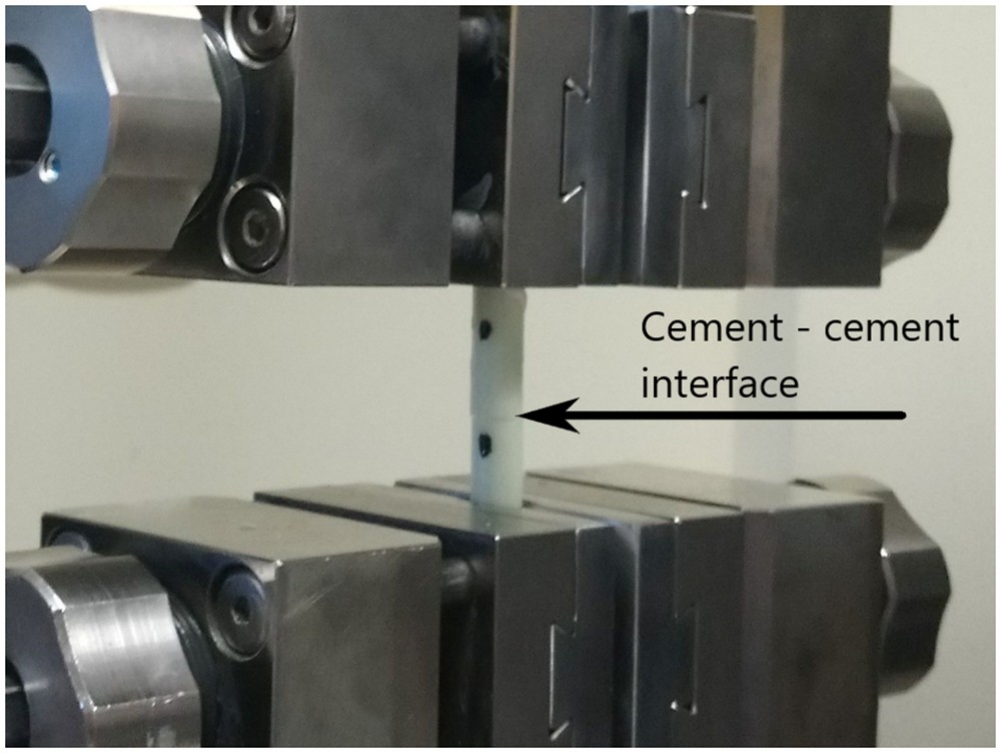
The testing of the composite cement sample.

The samples were tested at the Zwick Roell Z020 uniaxial testing machine (Figs 1 and 5).

**Fig 6:**
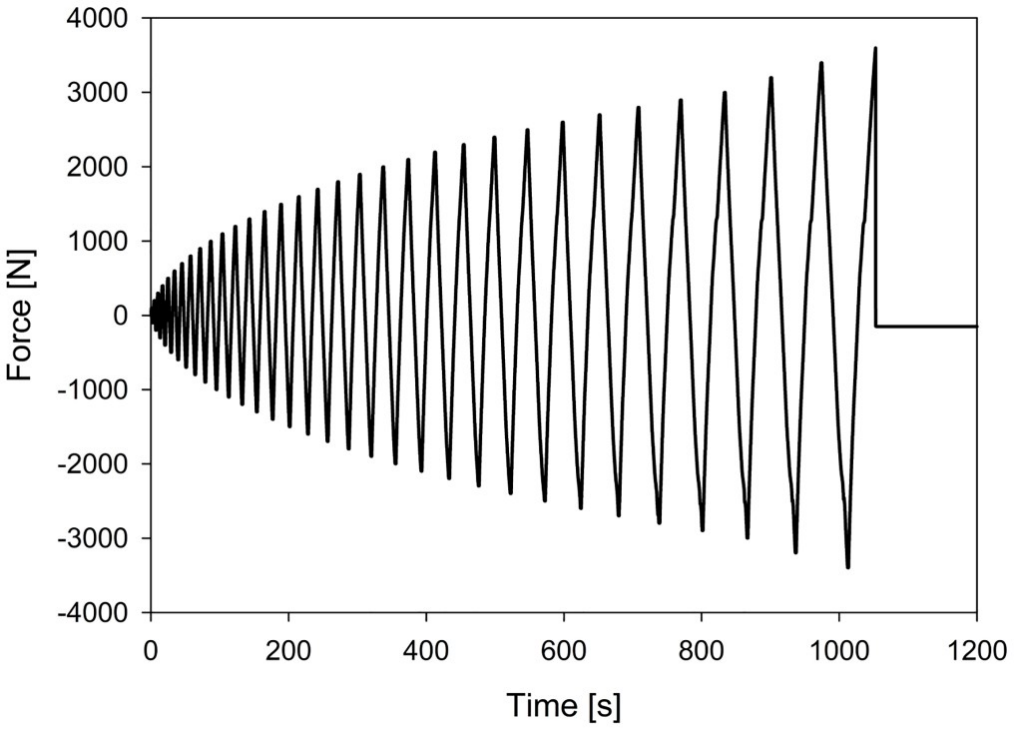
The force/time curve of the testing protocol.

The testing protocol was the fully reversed cyclic loading test—with increasing amplitude that included subsequent compression and tension phases (Fig 6). The preload was 50 N, the displacement rate was 5mm/min and the initial cycle loading was 100 N. In each subsequent cycle the load was increased by 100 N in both tension and compression phases until the force level reached 3000 N. After that, the force increment was automatically changed to 200 N. The procedure was continued till the fracture of the specimen.

The statistical evaluation was performed using Statistica 13.3 software. The Kolomgorov-Smirnoff test was used to assess the normal distribution. All samples results were normally distributed except for strength results for composite samples with 24 h polymerization time (*p* = 0.01). The ANOVA test was used for comparisons between groups. The T-Student test was used to individually compare between groups with normal distribution. For the individual comparisons that included the composite samples with 24 hours polymerization time the Mann-Whitney U test was used. Significance level was *p* < 0.05.

## Results

The results are given as force at sample failure for all tested specimens, as well as the failure stress of the connection in MPa (S1 File), in concordance with other studies [1]. All uniform cement samples fractured near the grips. All composite samples, both bone-cement and cement-cement fractured at the tested interface between bone and cement or the old and new cement.

The composite cement samples with 24 hour polymerization time were significantly stronger than bone-cement samples, as seen in Table 1 and S1 File (force at failure T-Student test *p < 0*.*001*; strength at failure Mann-Whitney U test *p = 0*.*02)*. There were no significant differences between the composite samples that polymerized for 24 hours before testing and those that polymerized for 6 weeks (force at failure: T-Student test *p = 0*.*93*; strength at failure Mann-Whitney U test *p = 0*.*35*).

**Table 1:** The comparison of force at failure (N) and strength at failure (MPa) between bone—cement samples and composite cement samples with 24 hour polymerization time.

	Composite cement sample (without drill hole) (*n* = 15)	Bone-cement sample (*n* = 31)
Force at failure (N)	902	284.16
	SD = 164	SD = 213.46
	Range: 577–1097	Range: 50–799
Mean strength at failure (MPa)	11.48	3.61
	SD = 2.09	SD = 2.71
	Range: 7.35–13.97	Range: 0.63–10.17

When comparing samples that polymerized for 6 weeks, the uniform cement sample was obviously the strongest of all specimens when comparing individually those samples and both types of composite samples, as seen in Table 2 and S1 File (T-Student test *p < 0*.*001*). All differences in these groups were significant in ANOVA test *(p < 0*.*001)*. The composite cement samples with drill hole on its surface were weaker than those without it (T-Student test *p = 0*.*046*).

**Table 2:** The comparison of force at failure (N) and strength at failure (MPa) of the samples with 6 weeks polymerization time.

	Uniform cement sample (control group) (*n* = 15) *S*_1_ = 78.54 mm^2^	Composite cement sample (without drill hole) (*n* = 22) *S*_1_ = 78.54 mm^2^	Composite cement sample (with drill hole) ^a^ (*n* = 16)	
			*S*_1_ = 78.54 mm^2^	*S*_3_ = 91.11 mm^2^
Mean force at failure (N)	3149.00	890.04	607.93	607.93
	SD = 418.48	SD = 561.45	SD = 355.35	SD = 355.35
	Range: 2097–3628	Range: 239–2278	Range: 198–1494	Range: 198–1494
Mean strength at failure (MPa)	40.09	11.33	7.74	6.67
	SD = 5.32	SD = 7.14	SD = 4.52	SD = 3.9
	Range: 26.69–46.19	Range: 3.04–29	Range: 2.52–18.89	Range: 2.17–16.28

^*a*^ The strength at failure values are given for the interface surface that does not account for the surface of the drill hole (*S*_1_ = 78.54 mm^2^) as well as for the surface that does account for it (*S*_3_ = 91.11 mm^2^), as described in the Materials and Methods section.

## Discussion

This study was designed to overcome some technical aspects that were criticized in previous studies. The bone samples were prepared as the third generation cementing technique requires: the bones were cleared with pulsatile lavage, the cement was prepared with an original vacuum system, applied under pressure and kept pressurized for 10 minutes. A long period of cement polymerization was ensured [1]. The cement surface was roughened with a rasp [21, 23], contaminated by human tissue as it would have been during real surgery [20] and wiped clean from macroscopic debris [5, 13, 21]. In the study no uncontaminated surfaces were used, as it is difficult to believe that during surgery they are perfectly clean as in laboratory testing. The novel element in this study is additional drilling of the old cement mantle, what should theoretically increase binding surface and provide superior anchoring of the old cement mantle [4]. The samples were cyclically loaded to make the experiment more similar to real life conditions, unlike in previous studies [28–30].

There are some limitations to this study. Shear forces more closely resemble forces found in femoral or humeral stem revision. In this study, for technical simplicity in manufacturing and for good reproducibility of the samples, the samples were constructed for tensile-compression strength testing only [20, 21, 24, 25, 31]. A clear limitation of both this and other biomechanical studies is that the cement interface was two-dimensional, and the old cement mantle is three-dimensional in vivo [1]. For that reason, and because hip and shoulder movement is three-dimensional, the forces that are applied to the revised arthroplasty component will be a combination of force vectors, and not a clear shear force only. However, most importantly, the tensile force and shear force tests show similar results: 80% and 85% of strength loss, respectively, therefore tensile testing still gives a good representation of the relative strength of different types of interfaces [20]. Moreover, tensile force testing is an accepted form of bone-cement interface strength [31].

In this study, cyclic loading was performed, what much better represents in vivo forces [1, 27]. Cyclic loading with force increasing by each cycle is an accepted and reliable form of cyclic testing in biomechanical studies [32, 33], with the advantage of being less time-consuming than standard cyclic loading tests of the constant force level [28–30].

The uniform cement samples fractured near the grips where the stress occurs concentration because of clamping, but the all the composite samples fractured at the connection interface, therefore these results of the study can be considered not confounded by the influence of the testing machine. The results shows the cement-cement bond, although much weaker than the uniform cement, is stronger than the bone-cement bond, assuming that old cement has mechanical and chemical properties similar to the new one [1]. Although the drill hole increases binding surface and theoretically provides additional anchoring in the old cement surface, the samples with a drill hole proved to be significantly weaker than those without it. The most probable explanation of this finding is that since the surface of the old cement was covered with fluid human tissues, as the surface of the old cement mantle would be intraoperatively [20], the fluids collected in the drill hole, from where they could not be removed by wiping of the surface with a gauze. When the new cement was introduced, the fluids were pressed out from the hole by the pressurized cement and formed a film on the surface of the old cement, thus limiting the contact surface between the old and the new part of the sample.

Overall, in all composite samples the strength of the connection is variable. The large differences between samples shows that the binding strength of the bone-cement and cement-cement interface may have significant local differences in actual revision arthroplasty [34].

The comparison of the results of this study with previous biomechanical studies is limited because significant differences in preparation of the samples and testing protocol.

A study by Dohmae et al. showed that the shear strength of bone-cement interface is reduced to 20.6% when the original cement mantle is removed and new cement is applied to the previously cemented femoral canal in first revision arthroplasty, and to only 6.8% in the second revision. This suggests that the original cement mantle, when not loose from the bone, may probably be still much stronger than any subsequent cementing of bone that is cleared of the original cement mantle. Unfortunately this study did not investigate the bond between old and new cement mantle, as the current study does.

A study of bone-cement interface used similar samples and tensile forces to test them [31]. The diameter of the samples was 12 mm v/s 10 mm in the current study, therefore the contact surface was 44% greater. The tensile force in that study was 492 N v/s 284 N in the current study (73% smaller force), what can be attributed both to the contact surface size, some differences in manufacturing of the samples or linear v/s cyclic testing.

Rosenstein et al. [25] performed a study comparing relative strength of bone-cement and cement-in-cement samples. The results supported the thesis that cement-in-cement is stronger than the bone-cement bond, in concordance with our study. The bone was damaged by complete removal of the old cement and the bone-cement interface strength was significantly reduced by 30% in revision bone samples compared to primary interfaces. The design of the study did not allow direct comparisons of strengths between those connections.

In Rosenstein et al., the “old” cement was tested after only 90 minutes, what is considered insufficient At least 24 hours of polymerization time of the cement is recommended before testing, since although initial polymerization and hardening of the cement occurs within 10 minutes after mixing of the components, but it continues for a much longer period of time [1]. A temporary decrease in bonding strength between days 0 to 14 occur of up to 19.4%, and then the strength of bilaminar cement mantles returns at day 30 to values similar to the ones at day 0 [24]. In the current study, the bone-cement samples were allowed only 24 hours polymerization time because to achieve a longer period of polymerization of bone-cement samples they would need to be stored either deep frozen with the risk of sample damage due to low temperature. The storage in temperatures of 4°C minimalized the risk of sample damage and bone decay. The temporary differences of cement strength were avoided by comparing the bone-cement samples to the group of cement samples that polymerized for 24 hours before testing. Moreover, no significant differences were found between samples that polymerized for 24 hours and those that polymerized for 6 weeks [24].

Li et al. [20] did not support the cement within cement revision. In this study, the surface of the old cement was contaminated with debris with no attempt to clear the contaminants from the surface of the old cement, contrary to what it is proposed to clear the cement surface meticulously during surgery [1, 21]. It is hypothesized that the debris or a film of fluid may prevent mechanical interlocking, but also may limit the chemical bond between the old and new cement [24]. Li et al. found a reduction of about 80% of strength of samples with contaminated interface, as compared with uniform cement mantle. An uncontaminated surface had only 11% reduction in strength. Greenwald et al. [21] found that the contaminated cement interface is weaker than uniform cement by only 37%. This is a smaller reduction than in Li et al. [20], perhaps because only blood and not debris or bone marrow was used.

In the current study, the strength of composite specimens showed a reduction of 72% (specimens without hole) and 84% (specimens with hole) of that of the uniform cement mantle. The reduction in strength of specimens without a hole was less than reported by Li et al. [20], most likely because in the current study the debris was wiped clean with a gauze what removed macroscopic debris and left only a film of fluid and perhaps some microscopic particles. The use of third generation cementing technique versus second generation probably played a role as well. Contaminating the interface with the freshly retrieved cancellous bone and not with blood, and wiping it clean more closely resemble the actual composition of the intraoperative contaminants than in other studies, as the surgeon will do their best to clean the cement surfaces from macroscopic debris [5, 13].

This study supports cement-in-cement revision arthroplasty. It should be concluded that it is better to leave the cement that is not loosened from the bone and perform cement in cement revision, than compromising bone stock by removal of the old cement. The bond between the old and new cement will still be stronger than the bond between cement and bone, and therefore it is theoretically less likely for loosening to occur at the cement-cement rather than on the bone-cement interface. The drill holes weaken the cement-cement connection, most likely because they collect fluid that cannot be reliably removed from it during surgery and which limit the binding of the new cement to the old one. Therefore drill holes in the old cement mantle are not recommended in this type of surgery.

## Supporting information

S1 File Data file. (XLSX)
